# Effects of mind-body exercise intervention on anxiety among women: a meta-analysis

**DOI:** 10.3389/fpsyg.2025.1652882

**Published:** 2025-09-29

**Authors:** Peng Chen, Yusha Gu, Nur Shakila Mazalan, Denise Koh, Weiping Du, Yuanyuan Luo

**Affiliations:** ^1^School of Physical Education, Ningxia Normal University, Guyuan, Ningxia, China; ^2^Faculty of Education, National University of Malaysia, Bangi, Selangor, Malaysia; ^3^Center for Sports and Health Research, Ningxia Normal University, Guyuan, Ningxia, China; ^4^Dazhou Vocational College of Chinese Medicine, Dazhou, Sichuan, China

**Keywords:** mind-body exercise, anxiety, women, female, mental health, effect size

## Abstract

**Objective:**

This meta-analysis aimed to evaluate the effectiveness of mind-body exercise (MBE) interventions in reducing anxiety among women and to explore potential intervention characteristics associated with greater efficacy.

**Methods:**

Seventeen studies involving 1,044 female participants were analyzed using Review Manager 5.3 and Stata 17.0. Subgroup analyses were conducted based on intervention type, weekly frequency, session duration, total intervention period, geographical region, and participant age. A random-effects model was applied to estimate pooled effect sizes and assess heterogeneity. The analysis adhered to Cochrane guidelines and was reported in accordance with the PRISMA checklist.

**Results:**

Mind-body exercise (MBE) interventions were associated with a significant reduction in anxiety symptoms among women, yielding a pooled standardized mean difference (SMD) of −1.14 [95% CI: (−1.56, −0.72), *p* < 0.00001]. However, substantial between-study heterogeneity was observed (I^2^ = 89%, Tau^2^ = 0.68), indicating considerable variability in effect sizes across studies. Among intervention types, Pilates showed the largest effect [SMD = −1.47, 95% CI: (−2.52, −0.41)], though this finding was based on only four studies and was accompanied by high heterogeneity (I^2^ = 93%), warranting cautious interpretation. Similarly, greater effects were observed for interventions involving 90-min sessions conducted three times per week over a period of 8–12 weeks [e.g., SMD = −1.46, 95% CI: (−2.18, −0.74)]. Nonetheless, these subgroup analyses also exhibited high heterogeneity (I^2^ values > 90%), suggesting that these parameters may not be universally optimal. Further subgroup analyses indicated stronger intervention effects in studies conducted outside China (SMD = −1.36, I^2^ = 93%) and among women aged 56 years and older (SMD = −1.30, I^2^ = 74%).

**Conclusion:**

Mind-body exercise interventions appear to have a substantial anxiolytic effect in women. However, these findings should be interpreted with caution due to the consistently high heterogeneity observed across analyses, as indicated by I^2^ values exceeding 85% in most subgroups and the presence of wide prediction intervals. Although certain formats, such as Pilates and intermediate-duration programs, show potential, further high-quality and culturally diverse trials are necessary to validate and refine intervention protocols.

**Systematic review registration:**

https://doi.org/10.37766/inplasy2025.6.0041.

## Introduction

Anxiety disorders are a leading cause of psychological distress worldwide, with women facing a significantly higher burden than men due to biological, psychological, and sociocultural factors (McLean et al., 2011). Biologically, susceptibility is rooted in estrogen fluctuations and genetic polymorphisms (e.g., COMT, 5-HTTLPR), which modulate neural reactivity to stress and emotional regulation (Schweizer-Schubert et al., 2021). Psychologically, cognitive patterns such as rumination (persistent negative self-reflection) and perfectionism exacerbate emotional dysregulation, fostering chronic anxiety through self-critical attribution of stressors (Flett et al., 2016). The main causes of female anxiety at the social level are the double burden of workplace difficulties caused by gender inequality and family work, as well as the industrialization of appearance anxiety and the intensified stereotypes of traditional gender roles (Powell, 2018). The issue of female anxiety has become increasingly prominent in today's society, and its negative impacts cannot be underestimated (Craske, 2003). Psychologically, anxiety can cause mood swings, insomnia, difficulty concentrating in women, and even increase the risk of mental illnesses such as depression (Zender and Olshansky, 2009). In terms of physical health, long—term anxiety is likely to trigger physical symptoms such as headaches, stomachaches, and endocrine disorders, and may also damage the cardiovascular and immune systems (Culpepper, 2009). These negative impacts severely reduce women's quality of life and pose a significant threat to their physical and mental health (Zender and Olshansky, 2009). While pharmacological interventions are widely used, their side effects and limited accessibility have spurred interest in non-pharmacological approaches, particularly mind-body exercise (MBE), which are defined as physical activities integrating somatic movement with mental regulation (Dong et al., 2024). This exercise typically includes various forms such as yoga, Tai chi, Pilates, and qigong (Chow et al., 2012). As a complementary and alternative therapy, MBE is prevalent among women due to its ease of learning and low requirements for equipment and space (Bandealy et al., 2021; Zhu et al., 2022). For example, yoga has been proven to be considered as a complementary therapy or an alternative method for medical therapy in the treatment of female anxiety disorders (Powell, 2018). Tai chi and Qigong exercise could reduce levels of anxiety and depression in those with Substance Use Disorder (SUB) (Zhang et al., 2022).

The anxiety-relieving effect of MBE on women is rooted in the synergy between physical engagement and mindfulness, which establishes a dynamically balanced “psycho-physiological” regulatory system (Rosenbaum, 2013). This system specifically targets and breaks the vicious cycle of “emotional amplification-somatic discomfort-cognitive rumination” characteristic of female anxiety (Kocsel et al., 2022). Psychologically, mindfulness-centered MBE diverts women's attentional focus from negative ruminative cognition to immediate somatic sensations, including respiratory rhythms and muscular tension (Payne et al., 2015). This process not only interrupts maladaptive anxious rumination, which is more commonly observed among women, but also facilitates self-acceptance through a non-judgmental approach, thereby enhancing self-efficacy and emotional regulation (Bernstein and McNally, 2018). Physiologically, MBE modulates stress through multiple neuroendocrine pathways (Jang et al., 2017). It downregulates hyperactivation of the hypothalamic-pituitary-adrenal (HPA) axis, thereby reducing cortisol secretion (Tafet and Nemeroff, 2020); stabilizes fluctuations in sex hormones, such as perimenopausal estrogen variability, which contribute to heightened anxiety sensitivity (Gordon et al., 2019); enhances parasympathetic nervous system activity, as indicated by increased heart rate variability (HRV) (Zou et al., 2018); and promotes the release of neurotransmitters such as gamma-aminobutyric acid (GABA) and serotonin, which play key roles in emotional regulation (Padmavathi et al., 2023). These physiological effects are particularly pronounced in women due to their inherently greater sensitivity of vagal tone and neurotransmitter receptors to stress (Kajantie and Phillips, 2006). Critically, psychological and physiological mechanisms interact dynamically: mindfulness-induced cognitive regulation weakens amygdala overactivation (reducing HPA axis activity) (Wheeler et al., 2017), while stabilized hormones and neurotransmitters alleviate somatic discomfort, reinforcing psychological control (Hall et al., 2023). This forms a “cognitive restructuring-neural regulation-somatic adaptation” cycle, underpinning MBE's efficacy in multidimensional anxiety relief for women (Mallorquí-Bagué et al., 2016).

Recent meta-analyses have examined the effectiveness of MBE interventions for anxiety management across a range of populations and clinical contexts. For example, Chandrababu et al. (2023) investigated the effects of yoga on anxiety, pain, and inflammatory and stress biomarkers in patients undergoing coronary artery bypass grafting (CABG) surgery in India, reporting significant psychological and physiological benefits. Similarly, Kraft et al. (2024) highlighted the potential of Taiji in alleviating perceived stress and improving depressive symptoms, anxiety levels, and physical quality of life in both clinical and healthy populations in Germany. In the United States, Taylor-Piliae and Finley (2020) demonstrated the efficacy of Tai Chi in promoting psychological well-being among individuals with cardiovascular disease. Complementing these international findings, several studies from China have also supported the benefits of MBE in managing anxiety and related symptoms. Zhu et al. (2021) analyze the effects of MBE on post-traumatic stress disorder (PTSD) symptom, depression and anxiety among patients with PTSD and to provide a scientific evidence-based exercise prescription. Dong et al. (2024) compare the clinical effects of different types of MBE on anxiety and depression in older adults. The results of the traditional meta-analysis showed that MBE were superior to the control group in alleviating anxiety (SMD: −0.87, 95% CI: −1.43, −0.31, *p* < 0.05, I-2 = 95%) and in the network meta-analysis, the ranking of treatment effects for anxiety showed that Tai Chi > Qigong > Yoga > Dance > control group. Li et al. (2020) analyze the effects of MBE for Chronic Obstructive Pulmonary Disease (COPD) patients with anxiety and depression and provide scientific evidence-based exercise prescription. SUB-group analysis indicated that, for anxiety, 30–60 min exercise session for 24 weeks of health qigong or yoga had a significant effect on patients with COPD who are more than 70 years and have more than a 10 years disease course. Xu et al. (2024) evaluate the impact of MBE, including tai chi, yoga, Pilates, qigong, baduanjin, and mindfulness-based stress reduction on anxiety among perimenopausal and postmenopausal women. Their findings demonstrated that MBE interventions yielded significant improvements in bone mineral density, sleep quality, anxiety, depression, and fatigue. While informative, Xu et al.'s analysis was limited to a hormonally specific subgroup and examined a broad spectrum of outcomes. In contrast, the present meta-analysis focuses exclusively on the anxiolytic effects of MBE across a more diverse female population, including women at various life stages. Furthermore, our study offers a detailed moderator analysis of key intervention parameters—such as session frequency, duration, total intervention length, and regional factors—to identify optimal delivery strategies for anxiety reduction. These aspects were not systematically explored in previous reviews. As such, the current study aims to refine and extend the existing evidence base by offering both theoretical and practical insights into the application of MBE for anxiety management in women.

Despite growing interest in MBE interventions for anxiety management, a significant limitation in the current literature is the reliance on gender-homogenized analyses, wherein data from male and female participants are often combined. This approach masks important sex-specific patterns in both psychological and physiological responses, particularly in women. Epidemiological data consistently show that women are nearly twice as likely as men to develop anxiety disorders across the lifespan (Hantsoo and Epperson, 2017), with this heightened vulnerability shaped by complex interactions among hormonal, neurobiological, and psychosocial factors. Biologically, women have a more responsive hypothalamic-pituitary-adrenal (HPA) axis and are more sensitive to fluctuations in sex hormones such as estrogen and progesterone, especially during menstruation, pregnancy, and menopause, all of which can exacerbate anxiety symptoms (Heck and Handa, 2019; Oyola and Handa, 2017). These endocrine factors also influence women's responsiveness to stress-reduction interventions (Carlson et al., 2004). For example, studies have shown that women tend to display stronger parasympathetic activation, greater interoceptive awareness, and more pronounced emotional regulation improvements during MBE practices such as yoga and tai chi, relative to men (Buric, 2018; Peng et al., 2024). Furthermore, although research on gender differences in stress physiology and coping styles is extensive, studies on key intervention parameters (e.g., optimal session duration, frequency, and total length of intervention) remain fragmented and largely gender-neutral. A targeted, female-centered research approach is therefore essential not only to elucidate mechanisms underlying MBE's anxiolytic effects in women but also to refine and personalize intervention protocols for enhanced clinical relevance and efficacy.

Although MBE has been increasingly recognized for its anxiolytic potential, the investigation of moderating factors remains methodologically limited. Specifically, previous research often fails to sufficiently account for cross-influences among exercise types, participant age groups, and sociocultural contexts. This limitation impedes a nuanced understanding of the mechanisms and boundaries through which MBE exerts its effects on anxiety in women. To address these gaps, the present meta-analysis aims to: (1) quantify the overall effect of MBE interventions on anxiety levels among women; (2) identify potentially effective intervention parameters; and (3) examine relevant moderators such as exercise type, geographic region, and participant age. By integrating evidence across culturally and methodologically diverse studies, this research seeks to provide empirically grounded recommendations for personalized MBE protocols and to contribute to the development of sex-specific anxiety interventions within the field of psychological health.

Utilizing a meta-analytic approach, this study evaluates the effectiveness of MBE interventions—excluding pregnant women and cancer patients—on female anxiety outcomes. The analysis incorporates specific intervention features (e.g., frequency, duration, modality) across a range of MBE types, including tai chi, yoga, qigong, and Pilates. The following hypotheses were proposed:

Hypothesis 1: Mind-body exercise interventions lead to a statistically significant reduction in anxiety symptoms among women.
Hypothesis 2: Of all MBE modalities, Pilates is associated with the largest reduction in anxiety symptoms.
Hypothesis 3: A frequency of three sessions per week produces stronger anxiolytic effects compared to other frequencies.
Hypothesis 4: A session duration of 90 min is associated with the greatest anxiety reduction.
Hypothesis 5: Intervention periods lasting 8–12 weeks yield more favorable outcomes for anxiety reduction.
Hypothesis 6: Studies conducted in countries outside of China report larger effect sizes for anxiety reduction among women compared to studies conducted within China.
Hypothesis 7: Women aged 56 and older exhibit greater anxiety reduction in response to MBE compared to younger age groups.

## Methodology

This meta-analysis was prospectively registered with INPLASY (Registration number: INPLASY2025.6.0041; doi: 10.37766/inplasy2025.6.0041).

### Acquisition and preliminary screening of literature

A comprehensive literature search was conducted across major Chinese and English-language databases, including the China National Knowledge Infrastructure (CNKI) for Chinese publications and Web of Science, PubMed, Cochrane Library, Embase, and Scopus for English publications. The search covered studies published from January 2000 to May 2025.

The search strategy employed two groups of keywords for both Chinese and English databases, combined using Boolean operators. For Chinese databases, the first keyword group included: “mind-body exercise,”“physical activity,” “movement,” “tai chi,” “yoga,” “qigong,” “pilates,” and “Baduanjin.” The second group comprised “anxiety” and “mental health.” For English databases, the first keyword group consisted of “exercises,” “physical exercise,” “physical activity,” “tai chi,” “yoga,” “qigong,” “pilates,” and “Baduanjin.”The second group included “anxiety,” “angst,” “nervousness,” “hypervigilance,” “social anxiety,” “anxiety social,” “social anxieties,” and “anxiousness.”

Keywords within each group were combined using the Boolean operator “OR,” while the two groups were connected using “AND.” The complete search strategy was first developed for PubMed and subsequently adapted for other databases. The search query used in PubMed is as follow:

(“anxiety”[MeSH Terms] OR “angst”[Title/Abstract] OR “nervousness”[Title/Abstract] OR “hypervigilance”[Title/Abstract] OR “social anxiety”[Title/Abstract] OR “anxiety social”[Title/Abstract] OR “social anxieties”[Title/Abstract] OR “anxiousness”[Title/Abstract]) AND (“exercise”[MeSH Terms] OR “physical exercise”[Title/Abstract] OR “physical activity”[Title/Abstract] OR “tai chi”[Title/Abstract] OR “yoga”[Title/Abstract] OR “qigong”[Title/Abstract] OR “pilates”[Title/Abstract] OR “Baduanjin”[Title/Abstract]) AND (“women”[Title/Abstract] OR “female”[Title/Abstract])

Meanwhile, the search query of Embase is as follow:

(‘anxiety':ti OR ‘angst':ti OR ‘nervousness':ti OR ‘hypervigilance':ti OR ‘social anxiety':ti OR ‘anxiousness':ti) AND (‘exercises':ti OR ‘physical exercise':ti OR ‘physical activity':ti OR ‘tai chi':ti OR ‘yoga':ti OR ‘qigong':ti OR ‘pilates':ti OR ‘baduanjin':ti) AND (‘women':ab,ti OR ‘female':ab,ti)

### Inclusion and exclusion criteria of literature

The literature screening adhered to the PICOS framework of evidence-based medicine, a systematic approach for evaluating research participants (P), intervention strategies (I), control/comparison methods (C), outcome metrics (O), and study designs (S) (Liberati et al., 2009). Eligible studies had to meet specific criteria: adopt a randomized controlled trial (RCT) design, recruit female participants, and include samples that fulfilled established diagnostic criteria for anxiety symptoms. The interventions were restricted to MBE, such as Pilates, Tai Chi, yoga, or Baduanjin, with each session lasting a minimum of 30 min and being administered multiple times throughout the intervention period. Control conditions were limited to non-exercise activities or maintaining routine lifestyles. Anxiety assessment had to be conducted using validated subjective measurement scales. Moreover, studies were required to report comprehensive data, including sample sizes, means, and standard deviations, for both the experimental and control groups.

Studies in the form of conference abstracts, case reports, or systematic reviews were excluded from the analysis. Additionally, studies involving participants with a history of regular physical exercise lasting more than 3 months, as well as those employing cognitive therapy as a control condition, were excluded based on the predefined criteria. Furthermore, studies with missing pre- or post-intervention data and those published in languages other than Chinese or English were also excluded from the present analysis.

### Literature screening and data extraction

**Figure 1:**
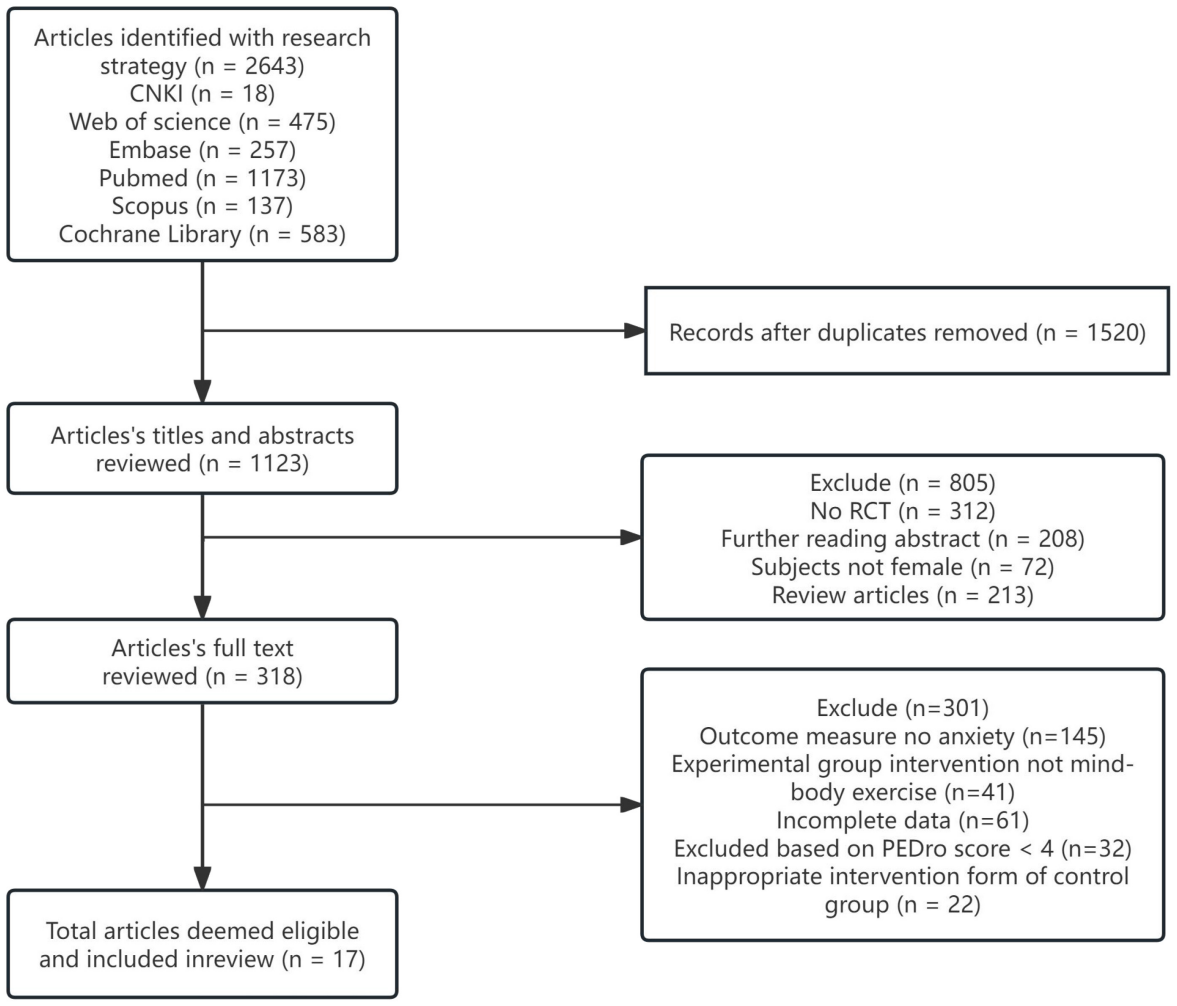
Flow diagram of literature selection.

The literature screening was conducted through a structured, sequential procedure. First, all retrieved bibliographic records were imported into the NoteExpress reference management system to remove duplicates. The primary author initiated the screening process by reviewing the titles, abstracts, and full texts of the identified publications. Subsequently, a second author independently evaluated the shortlisted articles to verify their compliance with the predefined inclusion criteria. In cases of disagreement, a third author was consulted, or the research team engaged in joint discussions to reach a consensus. This rigorous, multi-stage screening process ultimately yielded the final set of studies included in the meta-analysis, as illustrated in Figure 1.

PC and YG independently conducted data extraction in strict accordance with a pre-established standardized protocol. The extracted data included key elements such as the first author's name, publication year, study location, sample characteristics (including sample size and participant age), and comprehensive details of the intervention protocol, including intervention content, total duration, weekly session frequency, and individual session length. Additionally, outcome assessment measures were systematically collected. In cases where data were incomplete or unclear, the researchers made systematic efforts to contact the corresponding authors via email. If no response was received within 2 weeks, a follow-up email was sent. Studies with data that remained missing or ambiguous after these communication efforts were excluded from the final analysis to ensure data integrity and reliability.

### Literature review quality assessment

The methodological quality of the selected studies was appraised by applying the Physiotherapy Evidence Database (PEDro) scale. This scale consists of 11 items, with the first item not factored into the scoring process. For the remaining 10 items, a binary scoring system was employed: studies that satisfied the predefined criteria were awarded 1 point, while those that did not were given 0 points. Studies achieving a score of 6 or above were deemed to be of high quality. Two researchers independently evaluated each included article according to these PEDro scale guidelines. In instances where scoring discrepancies occurred, the disagreements were resolved either by consulting a third researcher or through collaborative discussions among the research team (Table 1).

**Table 1:** PEDro score of the included literature.

**Authors and year of publication**	**Eligibility criteria specified**	**Random allocation**	**Allocation concealment**	**Group similar at baseline**	**Blind subjects**	**Blind therapists**	**Blind assessors**	**Adequate follow-up**	**Intention to treat analysis**	**Intergroup statistical report**	**Point estimates and variability measures reported**	**PEDro score**
(Kang 2018)	1	1	0	1	0	0	0	1	1	1	1	6
(Zhang and Jiang 2023)	1	1	1	1	0	0	1	1	1	1	1	8
(Albracht-Schulte and Robert-McComb 2018)	1	1	0	1	0	0	0	1	1	1	1	6
(Gong and Zhang, 2019)	1	1	0	1	0	0	0	1	1	1	1	6
(Liu et al. 2025)	1	1	0	1	0	0	1	1	1	1	1	7
(Günebakan and Acar 2023)	1	1	0	1	1	1	1	1	1	1	1	9
Zhang J.-Y. et al. (2022)	1	1	1	1	0	0	1	1	1	1	1	8
(Armat et al. 2022)	1	1	0	1	0	0	0	1	1	1	1	6
(Aibar-Almazán et al. 2019)	1	1	1	1	0	0	1	1	1	1	1	8
(Alizadeh et al. 2023)	1	1	0	1	0	0	0	1	1	1	1	6
(Song et al. 2022)	1	1	1	1	0	0	1	1	1	1	1	8
(Chen 2017)	1	1	0	1	0	0	0	1	1	1	1	6
(MO 2016)	1	1	0	1	0	0	0	1	1	1	1	6
(Ma 2016)	1	1	0	1	0	0	0	1	1	1	1	6
(Shi 2022)	1	1	0	1	0	0	0	1	1	1	1	6
(Jia 2025)	1	1	0	1	0	0	0	1	1	1	1	6
(Akbaş and Ünver 2018)	1	1	0	1	1	0	0	1	1	1	1	7

1, criterion met; 0, criterion not met.

### Data analysis

The analysis of anxiety scale outcomes across selected studies was conducted using the random model in Revman 5.3 software. As the outcome measures were continue variables using consistent units, the standardized mean difference (SMD) was selected as the effect size metric. Effect sizes were interpreted according to (Cohen et al., 1983) guideline: SMD < 0.2 indicating negligible effect, 0.2 ≤ SMD < 0.5 representing small effect, 0.5 ≤ SMD < 0.8 denoting medium effect, and SMD ≥ 0.8 signifying large effect.

Study heterogeneity was evaluated using the *I*^2^ statistic. An *I*^2^ value of zero indicates homogeneity among studies, warranting the use of a fixed-effects model for effect size aggregation. Conversely, *I*^2^ values ≥ 50% suggest substantial heterogeneity, necessitating the application of a random-effects model and subsequent subgroup analyses to explore potential sources of variation.

## Results

### The basic characteristics of incorporated research literature

This study incorporated a total of 17 studies conducted in China, the United States, Turkey, Iran, Spain, and Malaysia, with a combined sample size of 1,044 participants. The sample encompassed a wide range of female populations, including female college students, postpartum women, breast cancer survivors, menopausal women, elderly women, and patients with diabetes or knee osteoarthritis. Participant ages ranged from young female college students (mean age: 19.16 ± 1.05 to 22.85 ± 1.26 years) to postmenopausal women (mean age: 69.98 ± 7.83 years). Sample sizes varied from 16 pairs (Turkish young women) to 55 pairs (Spanish postmenopausal women), with Chinese studies comprising the majority (12 out of 17 studies). All interventions were classified as mind-body exercises. In the experimental groups, the interventions included Pilates (4 studies), Yoga (7 studies), Qigong (4 studies), and Tai Chi (2 studies). Control groups generally adopted conditions such as “maintaining daily life” or “routine care.” Intervention durations varied widely, ranging from 4 weeks (e.g., a U.S. study on Yoga for female college students) to 24 weeks (e.g., traditional fitness Qigong for menopausal and elderly women in China). Individual session durations typically ranged from 30 to 90 min, conducted 2 to 3 times per week. Anxiety outcomes were assessed using a variety of standardized instruments, including the SCL-90, SAS, STAI, GAD-7, BAI, and HADS. Several studies employed composite scales such as the HADS to evaluate both anxiety and depressive symptoms concurrently (Table 2).

**Table 2:** Basic information of included literature.

**Author, year**	**Study location**	**subjects**	**Age E C**	**Sample size E C**	**Intervention measures E C**	**Time, frequency, duration**	**Outcomes**
(Kang 2018)	China	Female college student	Not mentioned	30 30	Pilates Maintain daily life	60 min 3 times/week 16 weeks	SCL-90
(Zhang and Jiang 2023)	China	Female college student	19.23 ± 0.98 19.16 ± 1.05	39 39	Qigong Maintain the original lifestyle	60 min 3 times/week 12 weeks	SCL-90
(Albracht-Schulte and Robert-McComb 2018)	American	Female college student	20.18 ± 1.97	20 20	Yoga Emotional picture stimulation	30 min 2 times/week 4 weeks	STAI
(Gong and Zhang 2019)	China	Female college student	22.85 ± 1.26	34 36	Yoga No regular exercise	40 min 3 times/week 8 weeks	SAS
(Liu et al. 2025)	China	Postpartum women	30.48 ± 5.36 30.45 ± 5.35	28 33	Yoga Maintain daily life	60 min 3 times/week 8 weeks	GAD-7
(Günebakan and Acar 2023)	Turkey	Healthy young adult woman	25.56 ± 4.55 29.81 ± 7.00	16 16	Yoga No sports training	45 min 2 times/week 6 weeks	STAI
Zhang J.-Y. et al. (2022)	China	Breast cancer survivor	47.79 ± 5.14 47.20 ± 7.65	29 29	Mindful Tai Chi Normal care	60 min 2 times/week 8 weeks	SAS
(Armat et al. 2022)	Iran	Retired women	57.7 ± 4.733 58.97 ± 5.368	30 28	Yoga Maintain daily life	90 min 2 times/week 8 weeks	BAI
(Aibar-Almazán et al. 2019)	Spain	Postmenopausal women	69.98 ± 7.83 66.79 ± 10.14	52 55	Pilates Maintain daily life	60 min 2 times/week 12 weeks	HADS
(Alizadeh et al. 2023)	Iran	Diabetic patients	20–55 years	30 30	Pilates Normal care	60 min 3 times/week 8 weeks	HADS
(Song et al. 2022)	China	Elderly woman with knee osteoarthritis	64.15 ± 8.56 64.15 ± 8.56	20 20	Improved Tai Chi Maintain daily life	60 min 3 times/week 12 weeks	SAS
(Chen 2017)	China	Menopausal women	50.6 ± 3.7	27 24	Qigong No regular exercise	30 min 3 times/week 24 weeks	SAS
(MO 2016)	China	Older women	65.7 ± 5.1 66.4 ± 4.9	39 40	Qigong No regular exercise	60 min 3 times/week 24 weeks	SCL-90
(Ma 2016)	China	Older women	60.9 ± 5.1 60.2 ± 4.1	38 40	Qigong No sports training	60 min 3 times/week 20 weeks	SAS
(Shi 2022)	China	Middle-aged women	52.76 ± 5.94 51.80 ± 3.28	30 30	Fitness Yoga Not participating in any sports	60 min 3 times/week 16 weeks	SAS
(Jia 2025)	China	Menopausal women	50.03 ± 2.87 49.52 ± 2.82	30 31	Yoga Not participating in any sports	90 min 3 times/week 24 weeks	SAS
(Akbaş and Ünver 2018)	Malaysia	Young women	21.44 ± 1.35 21.26 ± 1.51	25 26	Pilates Not participating in regular physical activity	40 min 2 times/week 6 weeks	BAI

E, Experimental group; C, control group; STAI, State–Trait Anxiety Inventory; SCL-90, Symptom Checklist 90, SAS, Self- rating anxiety scale; HADS, Hospital Anxiety and Depression Scale; BAI, Beck Anxiety Inventory; GAD-7, Generalized Anxiety Disorder 7-item scale.

### Publication bias test

**Figure 2:**
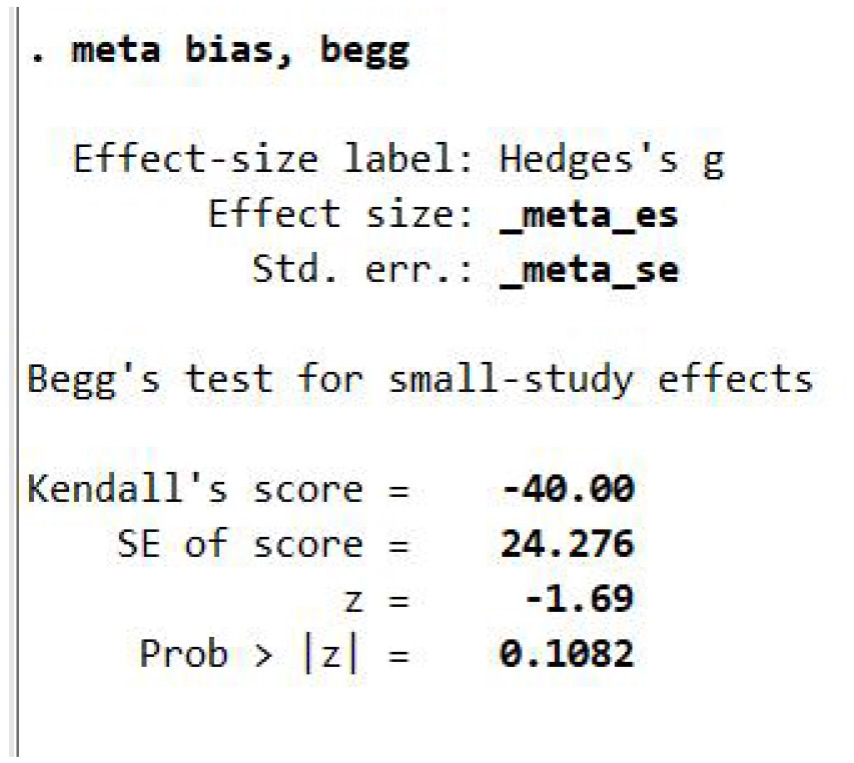
Begg test of publication bias.

The Begg's publication bias test was conducted using the meta bias, begg command in Stata, with the effect size labeled as “Hedges's g.”The analysis was based on the variables _meta_es (effect size) and _meta_se (standard error). The test yielded a Kendall's score of −40.00, with a standard error of 24.276, a corresponding standard normal z-value of −1.69, and a two-tailed *p*-value of 0.1082. Using the conventional significance level of 0.05, the *p*-value exceeds the threshold, and thus the null hypothesis cannot be rejected. This indicates that there is insufficient evidence to suggest the presence of publication bias in the current meta-analysis on the effects of MBE interventions on anxiety levels in women. In other words, no significant selective publication effects related to study outcomes were detected in the publication process of the included literature (Figure 2).

### Sensitivity analysis

**Figure 3:**
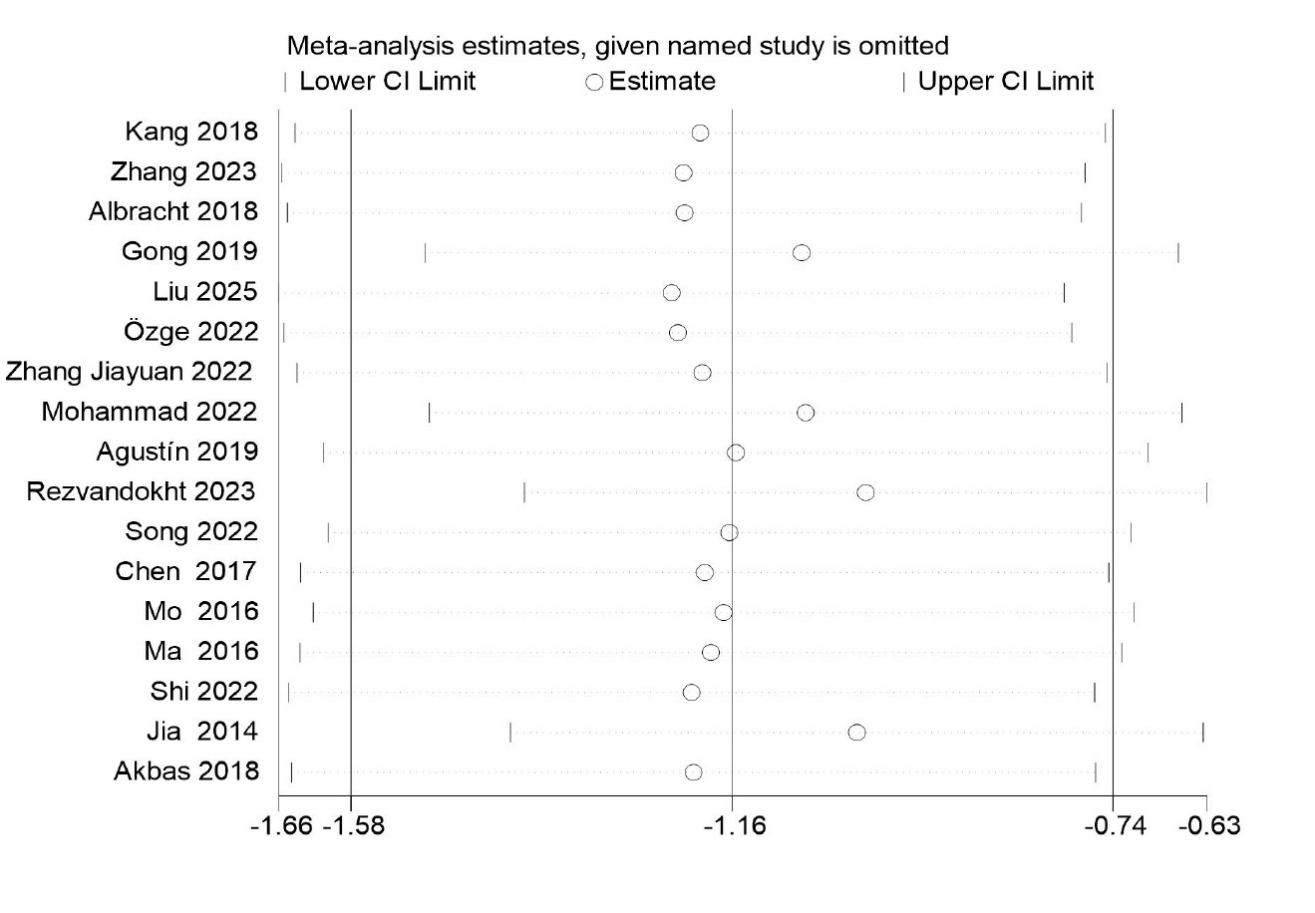
Sensitivity analysis.

Figure 3 presents the sensitivity analysis chart generated using Stata software, designed to assess the stability of the meta-analysis results concerning the effects of mind-body exercise interventions on anxiety levels in women. This analysis was performed by sequentially omitting each included study to examine its impact on the overall effect size. The horizontal axis displays the estimated range of the effect size along with its confidence interval, while the vertical axis lists the individual studies, including “Kang, 2018” and “Zhang and Jiang, 2023.” Each dot in the figure represents the recalculated effect size after removing the corresponding study, and the accompanying vertical line indicates the revised confidence interval, with its lower and upper limits marked accordingly. The central objective of this sensitivity analysis is to evaluate how strongly a single study influences the combined effect estimate. If the removal of any individual study results in only minor fluctuations in the effect size and its confidence interval, the meta-analytic findings are considered stable and exhibit low sensitivity to individual studies. As shown in the figure, the effect size estimates remain consistently centered around a relatively narrow range, regardless of which study is excluded. Additionally, the width and position of the confidence intervals show no substantial variation. These findings clearly indicate that the meta-analysis is not unduly influenced by any single study. Therefore, the sensitivity analysis supports the robustness and reliability of the meta-analytic results.

### Meta-analysis results

#### Overall effect test of intervention outcomes

**Figure 4:**
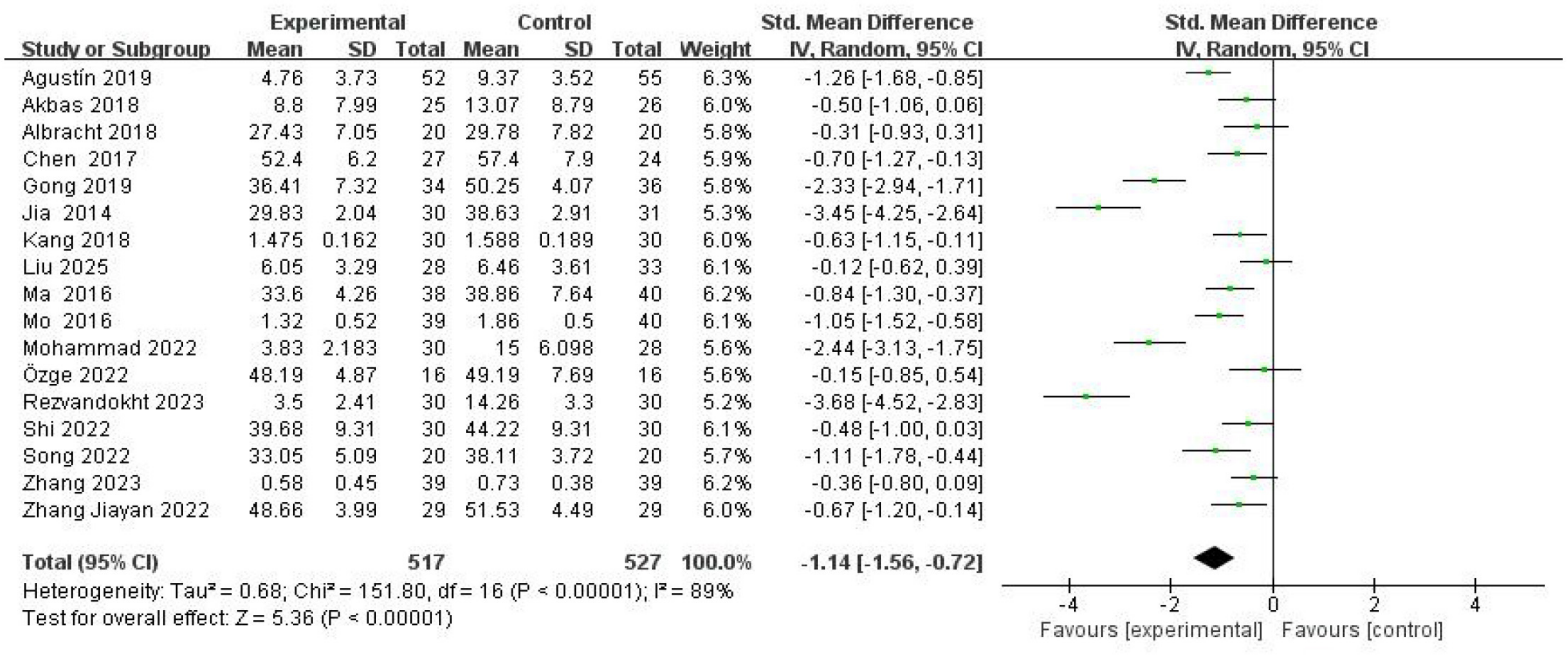
Forest plot of MBE interventions on anxiety in women.

A total of 17 literatures that met the criteria were included in this study for data integration (Figure 4). The results of the heterogeneity test showed that there was significant heterogeneity among the studies (chi-square test value: X^2^ = 151.80, *P* < 0.00001; heterogeneity index I^2^ = 89%), indicating that the differences in effect sizes between different studies far exceeded the scope of random errors, and a random effects model was required for combined analysis. The standardized mean difference (SMD) of the combined effect size was −1.14 (95% confidence interval: −1.56 to −0.72), indicating that mind-body exercise intervention can significantly reduce women's anxiety levels, and the absolute value of the effect size is greater than 0.8, which belongs to the large effect category. Statistical tests further confirmed the significance of the results (Z = 5.36, *P* < 0.00001), indicating that the intervention effect has robust statistical support. The sources of heterogeneity may be related to the diversity of intervention programs included in the studies (such as specific types of exercise such as yoga and Tai Chi), intervention periods (ranging from 4 to 12 weeks), sample characteristics (age range, differences in baseline anxiety levels), or assessment tools (such as different scales such as SAS and HAMA). Therefore, it is necessary to further study the moderating variables that may affect the overall treatment effect (Table 3). Thus, research hypothesis 1 has been verified.

**Table 3:** Overall effect of mind-body exercise intervention on anxiety.

**Quantity of literature**	**Heterogeneity test**			**Effect size and 95% confidence interval**	**Two-tailed test**	
	***X2***	***P***	***I2***		***Z***	***P***
17	151.80	< 0.00001	89%	−1.14 (−1.56, −0.72)	5.36	< 0.00001

#### Subgroup analysis of moderating variables

Given the substantial heterogeneity observed in the overall effect size analysis, subgroup analyses were conducted to investigate potential moderating variables. Six intervention parameters were examined as potential sources of heterogeneity: intervention type, weekly frequency, session duration, total intervention period, geographical region, and participant age (Table 4). These analyses aimed to systematically evaluate how variations in exercise program characteristics might influence intervention effectiveness.

**Table 4:** Results of moderating variable intervention on anxiety in exercise program.

**Moderating variable**	**Test of heterogeneity**			**Subgroup**	**Quantity of literature**	**Sample size**	**Effect size and 95% confidence interval**	**Two-tailed test**	
	***X2***	***P***	***I 2***					***Z***	***P***
Intervention type	43.36	< 0.00001	93%	Pilates	4	278	−1.47 (−2.52, −0.41)	2.73	0.006
	4.64	0.20	35%	Qigong	4	286	−0.73 (−1.03, −0.43)	4.76	< 0.00001
	1.05	0.31	4%	Taijiquan	2	98	−0.84 (−1.27, −0.41)	3.86	0.0001
	95.78	< 0.00001	94%	yoga	7	382	−1.31 (−2.24, −0.37)	2.74	0.006
Weekly frequency	32.65	< 0.00001	85%	2 times/week	6	346	−0.89 (−1.48, −0.29)	2.92	0.004
	118.78	< 0.00001	92%	3 times/week	11	698	−1.29 (−1.87, −0.71)	4.38	< 0.0001
Session duration	30.75	< 0.00001	87%	30–45 min	5	244	−0.80 (−1.56, −0.05)	2.08	0.04
	63.59	< 0.00001	86%	60 min	10	681	−0.96 (−1.40, −0.53)	4.35	< 0.0001
	3.45	0.06	71%	90 min	2	119	−2.92 (−3.91, −1.93)	5.81	< 0.00001
Total intervention period	0.61	0.74	0%	4–6 weeks	3	123	−0.35 (−0.70, 0.01)	1.90	0.06
	93.34	< 0.00001	93%	8–12 weeks	8	532	−1.46 (−2.18, −0.74)	3.96	< 0.0001
	42.52	< 0.00001	88%	16–24 weeks	6	389	−1.14 (−1.78, −0.50)	3.48	0.0005
Geographical region	79.42	< 0.00001	87%	China	11	696	−1.03 (−1.48, −0.57)	4.39	< 0.0001
	67.83	< 0.00001	93%	Foreign countries	6	348	−1.36 (−2.28, −0.45)	2.91	0.004
Participant age	37.61	< 0.00001	87%	19–31years old	6	332	−0.62 (−1.25, 0.01)	1.93	0.05
	41.77	< 0.00001	93%	47–55years old	4	230	−1.28 (−2.38, −0.19)	2.29	0.02
	15.27	0.004	74%	>56 years old	5	362	−1.30 (−1.76, −0.84)	5.55	< 0.00001

### Intervention type

The analysis of the intervention included 1,044 participants with a combined effect size of −1.14 [95% CI (−1.56, −0.72)], heterogeneity (I^2^ = 89%), (Z = 5.36), (*P* < 0.00001), which suggests that mind-body type of exercise interventions are overall effective in reducing women's levels of anxiety but that there is high heterogeneity across studies, which may stem from the population studied, differences in the details of the intervention program and measurement methods. Specifically, in subgroup analysis, Pilates involved 4 studies and 278 women, with an effect size of −1.47 [95% CI (−2.52, −0.41)], which was relatively large among the four types of exercise and had a significant effect on reducing women's anxiety levels. However, the heterogeneity of this subgroup was high (I^2^ = 93%), which may be due to differences in the characteristics of the research subjects, training content, etc., leading to large differences in results. A total of four studies were conducted, with 286 women participating in the Qigong exercises. The effect size was determined to be −0.73 [95% CI (−1.03, −0.43)], which was considered to be relatively small among the four exercises. However, it was observed that heterogeneity was low (I^2^ = 35%), and the consistency of the results was deemed to be good across the studies. This consistency can be attributed to the relative uniformity in the principles of the Qigong intervention and the modus operandi. Taijiquan included 2 studies, 98 women, and an effect size of −0.84 [95% CI (−1.27, −0.41)], with an intermediate effect size and very low heterogeneity (I^2^ = 4%), suggesting that the findings were highly similar, possibly due to the consistency of the taijiquan movement specifications and the flow of practice. The analysis revealed a significant effect size of −1.31 [95% CI (−2.24, −0.37)] for yoga, encompassing seven studies and 382 women. The study indicated a notable impact of yoga on reducing anxiety levels among women; however, it also exhibited substantial heterogeneity (I^2^ = 94%), potentially attributable to the wide spectrum of yoga genres, varying class content, and differing practice intensity and duration, which resulted in significant variations in study outcomes. Moreover, the findings of the subgroup differences test yielded Q = 2.82, df = 3, *P* = 0.42, I^2^ = 0%. Despite the presence of heterogeneity across subgroups, the overall difference between subgroups receiving distinct interventions was not significant. The findings were consistent across subgroups, exhibiting a high degree of reliability. This suggests the presence of variations in the efficacy of different mind-body exercise types in reducing anxiety levels in women. However, these variations may not be attributed to intrinsic differences in the exercise itself, but rather to random factors.

### Weekly frequency

A total of 1,044 participants were included in the subgroup analysis, categorized into two groups based on intervention frequency: twice per week and three times per week. In the twice-weekly intervention group, six studies were included, comprising a total of 346 participants. The test for heterogeneity revealed a high degree of heterogeneity (χ^2^ = 32.65, *p* < 0.00001, I^2^ = 85%). The pooled effect size was SMD = −0.89 [95% CI: (−1.48, −0.29), Z = 2.92, *p* = 0.004], indicating a statistically significant reduction in anxiety levels among women following twice-weekly mind-body exercise interventions.

In the three-times-weekly intervention group, eleven studies were included, with a total sample size of 698 participants. The heterogeneity test showed very high heterogeneity (χ^2^ = 118.78, *p* < 0.00001, I^2^ = 92%). The pooled effect size was SMD = −1.29 [95% CI: (−1.87, −0.71), Z = 4.38, *p* < 0.0001], also indicating a significant reduction in anxiety levels. Importantly, the larger absolute value of the effect size in the three-times-per-week group suggests that more frequent interventions may yield greater anxiety-reducing benefits. Nevertheless, the high heterogeneity observed in both subgroups suggests that these results may be influenced by unmeasured moderating variables (e.g., intervention type, participant characteristics, or study quality), and should therefore be interpreted with caution.

### Session duration

This analysis included 1,044 participants across three subgroups categorized by the duration of each intervention session: 30–45 min, 60 min, and 90 min. In terms of heterogeneity, the I^2^ values were 87%, 86%, and 71% for the 30–45 min, 60 min, and 90 min subgroups, respectively, indicating substantial between-study variability. Such heterogeneity may be attributed to differences in study design, participant characteristics, or implementation fidelity. Regarding effect sizes, the SMD for the 30–45 min subgroup was −0.80, suggesting a moderate effect in reducing anxiety among women. The 60 min subgroup yielded a slightly stronger effect (SMD = −0.96), while the 90 min subgroup demonstrated a markedly larger effect (SMD = −2.92), indicating a highly significant reduction in anxiety symptoms. The absolute values of the effect sizes followed the pattern: 90 min > 60 min > 30–45 min. The overall pooled effect size across all subgroups was SMD = −1.14, confirming the significant positive impact of mind-body exercise interventions on reducing anxiety in women. These findings suggest that, to a certain extent, longer intervention durations may be associated with greater therapeutic benefits, although the high heterogeneity observed warrants cautious interpretation.

### Total intervention period

This analysis included 1,044 participants, grouped into three subgroups based on the total intervention duration. In the 4–6 weeks subgroup, three studies with a total of 123 participants were included. Heterogeneity testing revealed Tau^2^ = 0.00, χ^2^ = 0.61, df = 2, *p* = 0.74, I^2^ = 0%, indicating negligible heterogeneity. The SMD was −0.35 [95% CI: (−0.70, 0.01), Z = 1.90, *p* = 0.06], suggesting a trend toward anxiety reduction, though not reaching statistical significance. The 8–12 weeks subgroup included eight studies with 532 participants. Heterogeneity was substantial (Tau^2^ = 0.99, χ^2^ = 93.34, df = 7, *p* < 0.00001, I^2^ = 93%), and the effect size was SMD = −1.46 [95% CI: (−2.18, −0.74), Z = 3.96, *p* < 0.0001], indicating a significant reduction in anxiety levels among women. In the 16–24 weeks subgroup, six studies with 389 participants were included. Heterogeneity remained high (Tau^2^ = 0.56, χ^2^ = 42.52, df = 5, *p* < 0.00001, I^2^ = 88%), and the pooled effect size was SMD = −1.14 [(95% CI: (−1.78, −0.50), Z = 3.48, *p* = 0.0005], again demonstrating a significant anxiolytic effect. The overall pooled effect size across all subgroups was SMD = −1.14 [95% CI: (−1.56, −0.72)], confirming the significant impact of mind-body exercise interventions in reducing anxiety among women. In terms of effect magnitude, the order of absolute effect sizes was: 8–12 weeks > 16–24 weeks > 4–6 weeks, suggesting that an intervention duration of 8 to 12 weeks may be the most effective within the observed range.

### Geographical region

This analysis included 1,044 participants, categorized into two subgroups based on the country in which the study was conducted: China and non-China. In terms of heterogeneity, the I^2^ value was 87% for the China subgroup and 93% for the non-China subgroup, both indicating high heterogeneity. This suggests considerable variability across studies within each region, potentially stemming from differences in sample characteristics, intervention protocols, or measurement instruments. Such variability may influence the accuracy and consistency of the observed effect sizes. Regarding the intervention effects, the SMD for the China subgroup was −1.03 [95% CI: (−1.48, −0.57), Z = 4.39, *p* < 0.0001], indicating that MBE interventions conducted in China produced a significant reduction in anxiety among women. This finding highlights the effectiveness of such interventions within the sociocultural, environmental, and demographic context of Chinese women. In the non-China subgroup, the effect size was even larger: SMD = −1.36 [95% CI: (−2.28, −0.45), Z = 2.91, *p* = 0.004], suggesting that MBE interventions outside of China were also significantly effective, with a greater magnitude of effect. This may imply that interventions conducted in non-Chinese settings yielded more pronounced improvements in anxiety, possibly due to regional differences in cultural attitudes toward exercise, support from healthcare systems for MBE, or pre-existing exercise habits among women. The overall pooled effect size across both subgroups was SMD = −1.14 [95% CI: (−1.56, −0.72)], further confirming the significant anxiety-reducing impact of MBE interventions in women. However, given the high heterogeneity observed in both subgroups, caution is warranted when interpreting and applying these findings. Regional differences and between-study inconsistencies should be carefully considered to ensure appropriate implementation and generalizability.

### Participants' age

In the subgroup analysis of participant age within the meta-analysis on the effects of mind-body exercise interventions on anxiety among women, differential results were observed across age groups. For the 19–31 years subgroup (6 studies, 332 participants), the SMD was −0.62 [95% CI: (−1.25, 0.01), *p* = 0.05], indicating an effect size near the threshold of statistical significance. This suggests a potential mild anxiety-reducing effect, although the result warrants cautious interpretation and further validation. The heterogeneity in this subgroup was high (I^2^ = 87%). In the 47–55 years subgroup (4 studies, 230 participants), the effect size was SMD = −1.28 [95% CI: (−2.38, −0.19), *p* = 0.02], which is both statistically significant and large in magnitude. This indicates a strong and reliable reduction in anxiety levels within this age range, though the heterogeneity was again substantial (I^2^ = 93%). For participants over 56 years of age (5 studies, 362 participants), the effect was the most pronounced, with SMD = −1.30 [95% CI: (−1.76, −0.84), *p* < 0.00001], reflecting a highly significant and strong anxiety-reducing effect. The heterogeneity for this group was moderate (I^2^ = 74%), suggesting relatively greater consistency across studies compared to the younger age groups. Overall, the pooled analysis demonstrated that mind-body exercise interventions significantly reduced anxiety levels in women, with a total effect size of SMD = −1.02 [95% CI: (−1.42, −0.62), *p* < 0.00001]. However, the consistently high heterogeneity across subgroups implies potential influences from varying intervention types, durations, or outcome measurement tools. These findings underscore the importance of tailoring intervention programs to specific age characteristics and highlight the need for further research to confirm the robustness and generalizability of these effects.

Up to this point, Hypotheses 1–7 have all been verified.

## Discussion

### Quality of included literature and overall effect size

Quality assessment of the 17 included studies yielded scores ranging from 6 to 9, with a mean score of 6.7, indicating generally high methodological quality. Lower quality scores were primarily attributed to insufficient reporting of randomization procedures, blinding protocols and participant attribution. Publication bias analysis revealed symmetric distribution of studies, suggesting robust stability of findings. The meta-analysis demonstrated a large significant effect of MBE interventions on anxiety reduction among women [d = −0.83, 95% CI (−1.18, −0.48), *p* < 0.001].

### Importance and significance of the research results

Although previous meta-analyses have investigated the effects of MBE on anxiety, they have generally focused on mixed-gender populations or specific clinical groups without disaggregating results by sex (Dong et al., 2024; Huang et al., 2023). In contrast, the present study is the first meta-analysis to systematically synthesize evidence exclusively on women, a population that exhibits significantly higher prevalence, earlier onset, greater chronicity, and increased comorbidity of anxiety disorders compared to men (Farhane-Medina et al., 2022). By isolating female participants across diverse intervention modalities, settings, and geographic contexts, this analysis delivers gender-specific insights that are both more actionable and clinically relevant. Building upon this gender-specific focus, the study further advances the literature by identifying optimal MBE intervention parameters tailored for women. Specifically, Pilates performed three times per week, for 90 min per session over 8–12 weeks, emerged as the most effective regimen—a level of prescription specificity seldom addressed in prior reviews. Such detailed recommendations provide practical guidance for clinicians, mental health professionals, and exercise instructors aiming to develop targeted, evidence-based interventions for female populations.

Additionally, subgroup analyses revealed that MBE interventions exerted particularly strong anxiolytic effects among older women (≥56 years), underscoring important age-related differences in responsiveness. This finding highlights the necessity of life-course–informed mental health strategies, especially for perimenopausal and postmenopausal women—a vulnerable and underserved group in psychological research (Miller et al., 2024). The study also explored geographic subgroup effects to examine cultural moderators of efficacy. While cultural differences did not significantly alter overall outcomes, these exploratory analyses underscore the potential influence of sociocultural contexts on intervention effectiveness and pave the way for future cross-cultural comparative research.

Central to this meta-analysis is its gender-responsive approach, which aligns with growing calls to move beyond simplistic male-female comparisons toward nuanced investigations of the biopsychosocial factors uniquely shaping women's emotional vulnerability and treatment response (Christiansen et al., 2022; Juster et al., 2016). Women's heightened anxiety risk is driven by the complex interplay of biological factors—including hormonal fluctuations across menstrual cycles, pregnancy, and menopause—psychological tendencies such as increased internalizing symptoms, and sociocultural pressures like caregiving demands and structural barriers to mental health care (Buric, 2018; Erving et al., 2022). These intersecting determinants not only elevate susceptibility but also influence how women respond to specific mind-body interventions. By explicitly acknowledging these layered influences, this study offers a refined understanding of the mechanisms through which MBE alleviates anxiety in women and supports the design of tailored interventions that address their unique physiological and psychosocial profiles (Andermann, 2010; Averill, 2015; Wood and Eagly, 2002). Taken together, this meta-analysis fills a critical gap in gender-specific mental health research and provides granular, evidence-based guidance to optimize MBE interventions for women across different ages and cultural backgrounds.

### Psychophysiological mechanisms of mind-body interventions

MBE interventions, such as Pilates, yoga, and tai chi, are believed to reduce anxiety through a multifaceted set of psychophysiological mechanisms (Lang, 2019). These mechanisms involve simultaneous modulation of the neuroendocrine system, autonomic nervous system, and central nervous system pathways implicated in emotional regulation (Critchley et al., 2013). The HPA axis plays a central role in the stress response system, and its dysregulation has been strongly associated with anxiety disorders (Faravelli et al., 2012; Tafet and Nemeroff, 2020). MBE help down regulate this system by reducing circulating cortisol levels, the primary stress hormone (Cahn et al., 2017; Chow et al., 2012). Studies have shown that regular engagement in yoga and tai chi significantly decreases cortisol secretion, promoting a return to homeostasis (Cahn et al., 2017; Yeung et al., 2018). Moreover, MBE promote parasympathetic nervous system activation, especially through breath-focused practices and slow movement (Wu et al., 2024). Increased vagal tone improves heart rate variability, a biomarker of emotional resilience and stress recovery (Weber et al., 2010). Techniques such as diaphragmatic breathing, used in Pilates and yoga, enhance vagal modulation and reduce sympathetic arousal (Adigüzel et al., 2023; Banushi et al., 2023). Anxiety is linked to heightened activity in the amygdala, a brain region involved in threat detection, and hypoactivity in the prefrontal cortex (PFC), which governs cognitive control (Kenwood et al., 2022). MBE are associated with increased PFC activation and decreased amygdala reactivity, improving top-down regulation of negative emotion (Zhang et al., 2019). Functional MRI studies have found that mindfulness-based exercise leads to increased gray matter volume in the PFC and anterior cingulate cortex, areas critical for emotion monitoring and regulation (Guendelman et al., 2017). These structural changes may underlie the long-term anxiolytic effects observed with consistent practice. Pilates and similar modalities emphasize body awareness, which enhances interoceptive accuracy, defined as the ability to perceive internal bodily sensations (Schleip and Jäger, 2012). Improved interoception has been linked to more adaptive emotion regulation and reduced anxiety (Pinna and Edwards, 2020). Regular practice fosters a mindful attention to physical sensations, which interrupts automatic negative thought patterns common in anxiety (Marchand, 2012). Mind-body practices also improve psychological constructs such as self-efficacy, self-compassion, and perceived control, which are important buffers against anxiety (Wong et al., 2021).

## Limitations of the research

This meta-analysis revealed substantial heterogeneity across the included studies, as indicated by high I^2^ values in several subgroup analyses. Such heterogeneity likely arises from differences in intervention modalities (e.g., yoga vs. Pilates), session frequency and duration, outcome measurement tools, and participant characteristics such as age, baseline anxiety severity, and overall health status. Although a random-effects model was employed to account for statistical variability, the extent of methodological and clinical diversity challenges the interpretability of the pooled effect sizes and limits the precision of the overall conclusions. Moreover, the lack of standardized intervention protocols and inconsistent reporting of key parameters—such as intervention intensity, adherence, and psychological context—further contributes to between-study variation. Future research should prioritize methodological rigor by standardizing intervention designs, adopting uniform outcome measures, and adhering to established reporting guidelines (e.g., CONSORT, PRISMA). Such improvements will facilitate more precise synthesis and improve the generalizability of future meta-analytic findings.

Approximately two-thirds of the included studies were conducted in China and predominantly recruited Chinese women. While this enhances sample homogeneity and strengthens internal consistency, it significantly limits the external validity and cross-cultural generalizability of the findings. Cultural context plays a pivotal role in shaping how women engage with MBE, perceive its effectiveness, and report psychological symptoms. However, the sociocultural influences on MBE engagement and symptom expression are complex and extend beyond a simple dichotomy of collectivist vs. individualist cultural frameworks. In collectivist societies such as China, women's participation in physical activity is often influenced by social values including modesty, family responsibility, and social harmony (Kim et al., 2025; Yang and Siu, 2025). These values may encourage MBE participation more as a social or relational obligation than for personal empowerment, which can in turn affect motivation, adherence, and subjective outcome evaluations. Conversely, in many Western cultures, MBE may be pursued more frequently as a path toward personal growth, emotional self-regulation, and autonomy (Box et al., 2019). Beyond this broad cultural dimension, other important sociocultural factors influence MBE engagement and psychological symptom reporting, such as religious beliefs, socioeconomic status, gender role expectations, health literacy, and stigma toward mental illness. For instance, spiritual or holistic health beliefs prevalent in some cultures may serve as significant motivators for participation, while economic barriers or limited mental health awareness may hinder engagement in others. Additionally, cultural norms shape how psychological distress is expressed; somatization of emotional difficulties, manifesting as fatigue or headaches, is common in many East Asian contexts (Wang et al., 2024), whereas in other cultures, emotional and cognitive symptoms may be more openly acknowledged and verbally expressed (Haft et al., 2022). These variations may impact self-report measures of anxiety and perceived effectiveness of MBE interventions, even when underlying physiological responses are comparable (Zou et al., 2018). Given this cultural complexity and heterogeneity, future research should move beyond using country of origin as a proxy for culture. Instead, studies should incorporate multidimensional cultural assessments—such as individualism–collectivism, emotion regulation styles, body awareness practices, and exercise-related beliefs—to more accurately evaluate how cultural factors moderate both the efficacy and acceptability of MBE interventions across diverse populations.

A notable limitation of this meta-analysis is the age distribution of participants across the included studies. Most trials targeted either college-aged women or postmenopausal populations, with a glaring absence of midlife women aged approximately 35 to 50. This underrepresentation is concerning from both clinical and theoretical perspectives, as midlife represents a critical period of heightened vulnerability to anxiety and stress-related disorders (Chourpiliadis et al., 2024; Li and Graham, 2017). During this stage, women experience a convergence of biopsychosocial stressors—such as perimenopausal hormonal fluctuations, intensified caregiving responsibilities, workplace role strain, and identity redefinition—that can exacerbate emotional instability (Mulhall et al., 2018). Moreover, midlife may represent a sensitive window in which tailored mind-body interventions could exert unique protective or restorative effects.

The current lack of developmental stratification prevents us from examining whether the efficacy of MBE varies across life stages or whether intervention protocols should be adapted to address the specific challenges of midlife. This limitation not only restricts the generalizability of our findings but also obscures important age-specific mechanisms through which MBE may operate. Future research should prioritize the inclusion of midlife women and adopt a developmental lens to better capture how anxiety manifests and responds to intervention across the female lifespan. Stratified recruitment and analysis by age cohort will help build a more comprehensive, equitable, and targeted evidence base for mental health intervention design.

## Discussion on heterogeneity and methodological rigor

Despite the substantial heterogeneity observed across the included studies, the results of the sensitivity analyses underscore the stability and credibility of the meta-analytic findings. The leave-one-out analysis, in which each study was sequentially excluded and the overall effect size recalculated, demonstrated that the pooled estimate remained relatively consistent, ranging from approximately −1.66 to −0.63. The central estimate exhibited no significant fluctuations, suggesting that no single study exerted disproportionate influence on the overall result.

In addition, influence diagnostics—including Baujat plots and Cook's distance—were employed to identify studies contributing most to heterogeneity and effect size variation. A few studies, particularly from the yoga subgroup (e.g., Aibar-Almazán et al., 2019; Akbaş and Ünver, 2018), showed relatively higher influence. However, their removal did not materially alter the direction or statistical significance of the pooled effect. These findings collectively affirm the robustness of the results and suggest that the conclusions are not driven by outliers or highly influential data points.

To further account for variability across studies, 95% prediction intervals were calculated alongside conventional confidence intervals. While confidence intervals indicate the precision of the pooled mean, prediction intervals estimate the range within which future study effects are expected to fall. In several cases, the prediction intervals were notably wide and occasionally crossed the null value. This implies that although the average effect was statistically significant, future implementations of mind-body interventions may yield variable outcomes. Such variability highlights the critical role of contextual and methodological factors—including participant characteristics, intervention fidelity, and cultural differences—in shaping treatment effectiveness.

## Future research directions

Given the limitations identified in this meta-analysis, several critical directions for future research are warranted to advance both the empirical robustness and practical utility of mind-body interventions (MBIs) for anxiety in women. First, future studies should address the substantial heterogeneity observed across existing trials. This includes standardizing key aspects of MBI protocols—such as type (e.g., yoga vs. tai chi), frequency, duration, instructor qualifications, and intensity levels—according to established frameworks like CONSORT and TIDieR (Howick et al., 2020). The use of uniform outcome measures and transparent reporting of adherence, psychological context, and intervention fidelity will improve comparability and enhance the interpretability of pooled effects. Second, to improve cross-cultural generalizability, future research should actively diversify participant populations beyond predominantly Chinese cohorts. Rather than using nationality as a proxy for culture, studies should incorporate multidimensional cultural indicators—such as individualism-collectivism, emotion regulation strategies, and exercise beliefs—alongside culturally validated instruments. Qualitative or mixed-method approaches may further illuminate how sociocultural values (e.g., body image, gender roles, stigma) mediate MBI uptake and perceived efficacy across cultural contexts. Third, addressing the current developmental gap is imperative. Midlife women (aged 35–50) remain markedly underrepresented, despite facing unique biopsychosocial stressors during this life stage—such as perimenopausal transitions, occupational strain, and familial caregiving demands—which may influence both vulnerability to anxiety and responsiveness to MBIs. Future research should intentionally include midlife women and adopt a lifespan developmental framework to examine whether intervention efficacy and mechanisms differ by age cohort. Stratified recruitment and analyses will allow for more personalized and age-sensitive mental health strategies. Finally, greater integration of objective, mechanistic assessments is essential. While most existing studies rely heavily on self-reported outcomes, future trials should incorporate biological markers (e.g., cortisol, heart rate variability), neuroimaging (e.g., EEG, fMRI), and behavioral indices to elucidate the underlying psychophysiological mechanisms of MBIs. This would foster theory-driven innovation and support the design of targeted, mechanism-informed interventions that can be tailored to individual needs across diverse female populations.

## Clinical recommendations

Based on the present meta-analytic findings, several practical recommendations can be made for clinicians, mental health practitioners, and exercise professionals seeking to integrate mind-body interventions into anxiety management strategies for women. First, MBE such as Pilates, yoga, and tai chi should be considered viable non-pharmacological options for women experiencing elevated anxiety symptoms. These interventions are low-cost, low-risk, and provide additional benefits for physical health, emotional regulation, and stress resilience, making them suitable for both clinical and community-based applications (Chawla et al., 2023; Kraemer et al., 2020). Clinicians are advised to tailor intervention plans to individual preferences, baseline fitness, and psychological readiness, especially across different female life stages. Midlife women, for instance, who are undergoing perimenopausal transitions and juggling caregiving and occupational responsibilities, may particularly benefit from interventions that promote hormonal balance, emotional grounding, and body awareness (Northrup, 2020). Subgroup findings from this meta-analysis suggest that optimal outcomes are associated with interventions lasting 8–12 weeks, involving approximately 90 minutes sessions held three times per week. Furthermore, culturally responsive strategies should guide the clinical implementation of mind-body interventions (DeLuca et al., 2018). In collectivist societies, women may be more receptive to group-based formats that foster interpersonal connectedness, shared purpose, and mutual accountability, which are factors known to enhance psychological safety and motivation in communal cultures (Ting-Toomey and Dorjee, 2018). Conversely, in individualist contexts, intervention engagement may be strengthened through flexible, self-directed formats that incorporate personalized feedback, autonomy-supportive coaching, and goal alignment with individual values (Ting-Toomey and Dorjee, 2018). Moreover, integrating structured psychological support, such as psychoeducation, mindfulness-based counseling, or motivational interviewing, can reinforce cognitive-emotional regulation, strengthen treatment adherence, and enhance the sustainability of outcomes (Berking et al., 2013). Such multimodal approaches align with transdiagnostic models of care and may be particularly beneficial for women with comorbid stress-related symptoms or limited prior exposure to therapeutic exercise (Wijnen et al., 2023).

## Conclusion

This meta-analysis synthesized data from 17 Chinese and English-language studies involving 1,044 women to examine the effects of mind-body exercise (MBE) interventions on anxiety. The overall results indicate that MBE can exert a significant and large effect on anxiety reduction in women. Among the various intervention types analyzed, Pilates demonstrated the largest standardized mean difference [SMD = −1.47, 95% CI (−2.52, −0.41)], but this finding was based on only four studies and was accompanied by substantial heterogeneity (I^2^ = 93%), calling for cautious interpretation.

Similarly, MBE interventions conducted three times per week for 90 minutes over a total duration of 8–12 weeks were associated with stronger effect sizes (SMD = −1.46), but again, this subgroup exhibited high heterogeneity (I^2^ = 93%) and wide confidence intervals. These results suggest that such parameters may be effective under certain conditions, yet they should not be considered universally optimal until further validated through higher-quality and more homogeneous studies.

Subgroup analyses revealed that intervention effects were particularly pronounced in studies conducted outside of China (SMD = −1.36) and among older women aged 56 and above (SMD = −1.30). These findings point to the potential importance of tailoring interventions based on sociocultural context and life stage. Personalized exercise prescriptions that take into account regional, cultural, and age-specific factors may enhance the relevance and efficacy of MBE interventions.

Nonetheless, considerable heterogeneity across studies—driven by differences in sample characteristics, intervention formats, and outcome measures—limits the generalizability of these findings. Therefore, practical application of the results should be adapted to individual and contextual factors. In conclusion, this meta-analysis provides evidence supporting the potential of mind-body exercise as a non-pharmacological approach to alleviating anxiety in women, while also highlighting the need for more rigorous, standardized, and culturally diverse research to refine and personalize intervention strategies.

## Data Availability

The original contributions presented in the study are included in the article/supplementary material, further inquiries can be directed to the corresponding author/s.
